# Evaluation of tourism competitiveness and mechanisms of spatial differentiation in Xinjiang, China

**DOI:** 10.1371/journal.pone.0263229

**Published:** 2022-02-07

**Authors:** Haijun Liu, Mihray Hasan, Dong Cui, Junjie Yan, Guojun Sun

**Affiliations:** 1 Institute of Resources and Ecology, Yili Normal University, Yining, China; 2 College of Biological and Geographical Sciences, Yili Normal University, Yining, China; 3 School of Environmental Law, Gansu University of Political Science and Law, Lanzhou, China; Northeastern University (Shenyang China), CHINA

## Abstract

Evaluation of tourism competitiveness is useful for measuring the level of regional tourism development. It is of great importance to understand the advantages and disadvantages of tourism development correctly and formulate corresponding development strategies. To investigate tourism competitiveness, this paper established an evaluation index system, including tourism development competitiveness, tourism resource competitiveness, and tourism-support competitiveness, for 14 prefectures and cities in Xinjiang in China. The characteristics and laws of spatial differentiation were analyzed. Factor analysis was applied to examine the spatial differentiation of regional tourism competitiveness. The results showed an obvious spatial differentiation in tourism competitiveness among the 14 prefectures and cities. In terms of development competitiveness, Yili and Urumqi constituted the spatial center, followed by Changji, Altay, and Ba Prefecture. As the provincial capital, Urumqi has political, economic, cultural, transportation, and geographic advantages, but its competitiveness is not prominent in terms of monopoly and efficiency. In terms of resource competitiveness, Yili is the core attraction, while Urumqi, Kashgar, Altay, and Ba Prefecture are dominant attractions. With respect to supporting competitiveness, Bo Prefecture has high value, followed by Urumqi City and Aksu. Hetian and Ke Prefecture have the lowest values. The comprehensive competitiveness of tourism is centered on Yili. Urumqi and Bo Prefecture are subcenters, and Changji, Altay, Ba Prefecture, Aksu, and Kashgar are characterized as multi-polar competition areas. Using the KMO and Bartlett’s sphericity tests, the cumulative contribution variance of the eigenvalues of the eight factors extracted by the maximum variance rotation method was found to be 92.714%. Socio-economic conditions, tourism resources, infrastructure construction, regional cultural influence, ecological environment carrying capacity, tertiary industry development, tourism service level, and living security system are the main driving factors affecting the spatial differentiation of tourism competitiveness in Xinjiang. Analyzing the spatial evolution characteristics and the driving factors of the regional tourism competitiveness in Xinjiang, this paper seeks to promote the optimal allocation of tourism production factors in the macro regional system, and provide theoretical guidance and an empirical basis for the comprehensive and harmonic development of regional tourism.

## 1. Introduction

In recent years, increasing attention is being paid to the economic and social benefits created by the tourism industry, a pillar industry for many regions [1]. The study of the regional tourism economy is important for grasping the opportunities for tourism development, tapping the potential of tourism resources, and enhancing the competitiveness of the tourism market [2, 3]. However, due a division of regional interests stemming from regional economic concerns has led to competition in tourism between different regions that is becoming increasingly fierce [4, 5]. It is important to measure and analyze the level of regional tourism competitiveness to correctly understand relative advantages and disadvantages in tourism development, the formulation of regional tourism development strategies, and the promotion of improved participation in domestic and foreign tourism market competition to accomplish the sustainable development of regional tourism [6].

Experts and scholars began to study the issue of tourism competitiveness in the 1990s. Porter put forward the Diamond Model of industrial international competitiveness involving production factors, demand status, business strategy, competition mode, auxiliary industries, opportunities, and government behavior, which laid a theoretical foundation for the study of system competitiveness [7]. Crouch et al. created a precedent in the study of tourism competitiveness by building a competitiveness evaluation system for tourism destinations with the diamond model [8]. Soon afterwards, scholars expanded their research perspective from the source competition of the tourism destination to a variety of factors that affect the competitiveness of tourism destinations, such as tourism products, price, transportation, environment, sustainable development, and so on. This study focuses on the concept of tourism competitiveness, which is defined from the perspective of market position and market share [9], sustainable development of tourism resources [10–12], increasing product value [13], and destination attraction, among other determinants [14]. The connotation of tourism competitiveness includes the economic ability of the competition subject and the sustainable development ability of tourism, but in general, it comes under the category of economic significance. The earliest examples of tourism competition evaluation models are the tourism destination competitiveness model [15], which was established based on the principles of consumers, product quality, system innovation, participant status, and house-type structure model [16]; the comprehensive system model of tourism resources [17]; and comprehensive model of destination competitiveness [18]. These models are constantly supplemented and improved with the development of the connotation of tourism competitiveness and the application of models under different time and space conditions. For evaluating tourism competitiveness, qualitative analysis methods and combining qualitative and quantitative analysis, such as performance analysis, analytic hierarchy process, partial least squares, factor analysis, data envelopment analysis, and panel data analysis have been used [19–23]. The study of influencing factors of tourism competitiveness are generally focused on the perspectives of marketing [24], tourism supply and demand system [25], environmental factors [26, 27], human resources and management [28], technological innovation [29], laws, and policy frameworks [30, 31].

Study of tourism competitiveness is relatively recent development in China. *The International Competition Strategy of China’s Tourism Industry*, written by Senlin Yang and others in 1996, was a prelude to the study of China’s tourism competitiveness [32]. Theoretical and empirical research on the concept and connotation, evaluation index systems, evaluation methods, influencing factors, and promotion strategies from scales or perspectives of countries, regions, cities, and tourism enterprises concerned the experts and scholars in China the most [33].

The research concept and connotation mainly includes competitiveness in tourist destinations [34], the spatial competitiveness of tourist attractions [35–37], competitiveness of the regional tourism industry [38], and competitiveness of urban tourism [39–41]. The theoretical research in this context mainly draws lessons from theories of other disciplines. Theoretical models of management, economics, geography, and sociology, among other disciplines, have been continuously applied to the evaluation of tourism competitiveness; examples include comparative advantage theory, competitive advantage theory, Porter’s diamond model, and so on [42–44]. In terms of research methods, the initial stage is mainly focused on the qualitative perspective. At a later stage, primarily methods of quantitative analysis were used, including analytic hierarchy process, coefficient of variation, factor analysis, principal component analysis, system clustering, neural network analysis, and geospatial analysis [45–49]. For the study of tourism competitiveness, in the initial stages, natural resource endowment and production factors, in the later stages, a multi-perspective of society, economy, culture, political environment, and the tourism industry itself were accepted and adopted. This multi-perspective method focuses on transforming the advantages of tourism resources into products or competitive advantages [50–53]. The early stages focus on how to develop the international market and meet the demands of international consumption. Then, the focus is transferred to the domestic tourism market in China and the countermeasures to improve tourism competitiveness from multiple angles [54–57].

Experts and scholars gradually formed a relatively systematic and complete body of results regarding tourism competitiveness. Although differences were found in research perspectives, methods, contents, and paths, they mainly focus on connotation definition, theoretical models, research methods, driving factors and promotion strategies. All these studies have produced influential perspectives in this academic field and greatly enriched the theoretical treasure house of tourism research. However, some research remains to be done. For instance, most research hitherto has been performed on the national or provincial scale. Studies on the medium and micro scales are still lacking. The purpose of tourism competitiveness evaluation is to accurately identify the advantages and disadvantages of regional tourism and provide theoretical support and empirical basis to formulate development strategies accordingly. From the perspective of research methods, the evaluation indicators cannot reflect the development level of tourism objectively. The negative factors affecting tourism development and the negative effects of tourism development are often ignored. Different research methods usually lead to subjective weight assignment of the index factors and gaps between evaluation results and the actual situation. Therefore, we should consider the construction of an index system including economic, social, ecological, and other dimensions based on a hypothesis that coordinates both the human development of land exploration, and try to use an objective weighting method in the data process to avoid the interference of subjective cognizance. This paper constructed an evaluation index system that takes the three aspects of development competitiveness, resource competitiveness, and support competitiveness into account. Then the spatial characteristics and driving factors for the comprehensive competitiveness of the 14 prefectures and cities in Xinjiang, China, were analyzed with the methods of entropy weighting and factor analysis. This paper promotes the optimal allocation of tourism production factors in the macro regional system and provides theoretical guidance and empirical basis for the coordinated development of regional tourism economy.

## 2. Overview of research area

Xinjiang is located at the center of the Eurasian continent, at the northwest border of China, ranging from 73° 40’ to 96° 18’ E and from 34° 25’ to 48° 10’ N, with an area of about 1 664 900 km^2^, accounting for about one-sixth of China’s land area. It is the largest Provincial Administrative Region in China. Administrative divisions include 4 prefecture-level cities; 5 regions; 5 autonomous prefectures; 13 municipal districts; 26 county-level cities, 61 counties, and 6 autonomous counties. The research area of this paper covers 14 prefectures and cities in Xinjiang, excluding Xinjiang Production and Construction Corps and Corps-organized cities. Xinjiang is important for the development of China’s tourism industry due to its vast territory, geographical advantages, and rich tourism resources. During the 13th Five Year Plan period, the development of Xinjiang’s tourism industry was focused on the strategy termed Silk Road Economic Belt and 21st Century Maritime Silk Road, which took the historical opportunity to pursue tourism assistance to Xinjiang and build a tourism distribution center of the Silk Road Economic Belt and Southern Xinjiang’s Silk Road culture and ethnic customs tourism as a destination. As of 2020, there are 501 A-level scenic spots in Xinjiang, including 12 5A level scenic spots, 109 4A level scenic spots and 287 3A level scenic spots. It also hosts 48 national wetland parks, 113 national heritage sites and 371 national intangible cultural heritage sites. Tourism is the pillar industry of Xinjiang and plays an important role in the economic development of the region, adjusting its industrial structure and increasing employment.

## 3. Data sources and research methods

### 3.1. Data sources

The data used in this paper are from the *Xinjiang Statistical Yearbook*, *China Tourism Statistical Yearbook*, and *China Urban Statistical Yearbook*, as well as from the *Xinjiang National Economic and Social Development Statistical Bulletin* and the tourism official website of Xinjiang (http://wlt.xinjiang.gov.cn/wlt/xxgk/xxgk.shtml), along with *The 13th Five Year Plan of Xinjiang Uygur Autonomous Region Tourism Development*.

### 3.2. Research methods

#### 3.2.1. Entropy method

The dimensions of various indicators in the original data matrix are different; further, the same indicator has different values in different units, which cannot thus be directly used for comparison and calculation. Therefore, it is necessary to standardize the original data for multi-attribute decision-making to eliminate the impact of indicator type and unit dimension on the result of the evaluation. In this paper, the range standardization method is used to deal with the original data.

We set *x*_*ij*_ as the index value of item *j* (*j* = 1, 2,…, *n*) in the *i* (*i* = 1, 2,…, *m*) evaluation unit, then

$X_{ij}^{+}$ refers to the positive indicator, and the standardized treatment is $x_{ij}^{‘}=\frac{X_{ij}^{+}-\underset{1\leq i\leq m}{\min}X_{ij}^{+}}{\underset{1\leq i\leq m}{\max}X_{ij}^{+}-\underset{1\leq i\leq m}{\min}X_{ij}^{+}}$

$X_{ij}^{-}$ refers to the negative indicator, and the standardized treatment is $x_{ij}^{‘}=\frac{\underset{1\leq i\leq m}{\max}X_{ij}^{-}-X_{ij}^{-}}{\underset{1\leq i\leq m}{\max}X_{ij}^{-}-\underset{1\leq i\leq m}{\min}X_{ij}^{-}}$

The concept of entropy originated in physics and was then introduced to information theory by Shannon. The entropy method can overcome the subjectivity of artificially determined weightings and the overlap of information among multi-index variables in complex giant systems. It is widely used in studies of social economy and similar research. The entropy method can reflect the utility value of index information entropy, and the predicted index weight has higher reliability, making it suitable for comprehensive evaluation of multiple indexes [58]. Because regional tourism competitiveness is a giant and complex system, the entropy method can be used to solve the problem of subjective weighting of randomness.

The specific steps in the calculation are as follows:

1. On the basis of 2.2.1. data standardization, we calculate the proportion of the *j* index value in the *i* evaluation unit: $y_{ij}=x_{ij}^{‘}/\sum_{i=1}^{m}x_{ij}^{‘}$
2. Entropy of the *j*-th index: $e_{j}=-k\sum_{i=1}^{m}y_{ij}\times lny_{ij}$, (*k =* 1*/*ln*m*), then $e_{j}=(-1/lnm)\sum_{i=1}^{m}y_{ij}\times lny_{ij}$
3. Coefficient of variance for the *j*-th index: *g*_*j*_ = 1−*e*_*j*_
4. The weight of the *j*-th index *w*_*j*_: $w_{j}=g_{j}/\sum_{i=1}^{m}y_{ij}\times lny_{ij}$
5. Calculate the comprehensive evaluation value *F* of tourism competitiveness of each evaluation unit using the weighted summation method: $F=\sum_{j=1}^{n}w_{j}\times x_{ij}^{‘}$

#### 3.2.2. Factor analysis

Factor analysis identifies a few variables to synthesize all variables by studying the internal dependence of the correlation coefficient matrix among the variables, grouping them according to their level of the correlation to produce a higher correlation between variables in the same group and a lower one between variables in different groups [59].

For *N* samples, *P* indicators, random vector *X* = (*X*_1_, *X*_2_,⋯,*X*_*p*_)′, and common factor *F* = (*F*_1_, *F*_2_,⋯,*F*_*m*_)′, the model could be written as follows:

$$X_{1}=\alpha_{11}F_{1}+\alpha_{12}F_{2}+\cdots +\alpha_{1m}F_{m}+\varepsilon_{1},$$

$$X_{2}=\alpha_{21}F_{1}+\alpha_{22}F_{2}+\cdots +\alpha_{2m}F_{m}+\varepsilon_{2},$$

⋮

$$X_{p}=\alpha_{p1}F_{1}+\alpha_{p2}F_{2}+\cdots +\alpha_{pm}F_{m}+\varepsilon_{p},$$

Here, matrix {*α*_*ij*_} is the factor load matrix, *α*_*ij*_ is the factor load, and *ε* is an influencing factor other than the common factor. With the rotation of the factor axis, the values of each element in the load matrix are differentiated to 0–1, such that the load of each variable on the factor is more obvious.

### 3.3. Construction of an index system

The study orientations of regional tourism competitiveness can be grouped into two sets of views. The first is the view of performance. Scholars who hold this view take account of the strength of regional tourism competitiveness according to its performance and consider that tourism competitiveness represents the ability of the tourism of the region to develop. The second is the factor view. Scholars who hold this view investigate the driving factors of tourism competitiveness, which can indicate its influencing mechanism while measuring tourism competitiveness. The evaluation of tourism competitiveness enables an understanding of the competitiveness of regional tourism industry and an exploration of its advantages and disadvantages; clarifies the focus and direction of tourism competitiveness cultivation; and aids in formulating effective competitive strategies to improve the overall competitiveness of the regional tourism industry. The two viewpoints each have their own emphases and cannot be judged simply. It is also inevitable that such assessments will be incomplete because of being based on a single orientation. Taking the above considerations into account and following the principles of the scientific approach, comparability, representation and availability of data, this paper combined the two views, established a conceptual framework, and set up indexes accordingly [60]. Three aspects of tourism competitiveness were taken into account in this index system, namely, tourism development competitiveness, resource competitiveness, and support competitiveness. Tourism development competitiveness is oriented by the performance view, while tourism resource competitiveness and tourism-support competitiveness are oriented by the factor view [61]. The index system is generally divided into four layers: the target layer reflects the comprehensive competitiveness of regional tourism; the criterion layer decomposes the target layer, including tourism development competitiveness, resource competitiveness, and support competitiveness; the factor layer refines the criterion layer; and the index layer is the specific evaluation factor of regional tourism competitiveness, including 35 factors (Table 1). Among the 35 factors, *X*_1_, *X*_2_, *X*_3_, *X*_4_, *X*_8_, *X*_9_, *X*_20_, and *X*_21_ are from the official website of Xinjiang tourism (http://wlt.xinjiang.gov.cn/wlt/xxgk/xxgk.shtml). *X*_5_, *X*_6_, *X*_7_, *X*_14_, *X*_18_, *X*_19_, *X*_22_, *X*_23_, *X*_25_, *X*_29_, *X*_31_, and *X*_33_ are from the *Xinjiang Statistical Yearbook*. *X*_10_, *X*_11_, *X*_12_, and *X*_13_ are from the *List of A-level Scenic Spots in Xinjiang*. *X*_15_ and *X*_16_ are from the official website of the State Administration of Cultural Relics of China (http://nb.ncha.gov.cn/). *X*_17_ is from the *List of China’s National Intangible Cultural Heritage*. *X*_24_, *X*_26_, *X*_27_, and *X*_28_ are from the *Xinjiang National Economic and Social Development Statistical Bulletin*. *X*_30_ is from the Meteorological Bureau of Xinjiang Uygur Autonomous Region (http://xj.cma.gov.cn/qxfw/fzjz/). *X*_32_, *X*_34_, and *X*_35_ are from the *China Urban Statistical Yearbook*.

**Table 1:** Evaluation index system of tourism competitiveness.

Target Layer	Criterion Layer	Factor Layer	Indicator Layer	Unit	Weight
TC	TDC (weight 0.4296)	Tourism Revenue	Tourism foreign exchange income *X*_1_	ten thousand yuan	0.2141
			Domestic tourism revenue *X*_2_	ten thousand yuan	0.1253
			Number of guest rooms *X*_3_	pcs	0.0755
			Number of beds in guest rooms *X*_4_	pcs	0.0764
		Economic Development Strength	Total GDP *X*_5_	ten thousand yuan	0.0828
			Per capita GDP *X*_6_	yuan	0.0561
			Proportion of tertiary industry in GDP *X*_7_	%	0.0977
		Tourism Development	Number of international tourists *X*_8_	ten thousand people	0.1913
			Number of domestic tourists *X*_9_	ten thousand people	0.0809
	TRC (weight 0.2283)	Tourist Resources	Total number of class A attractions *X*_10_	pcs	0.0436
			Total number of class 5A attractions *X*_11_	pcs	0.0883
			Total number of class 4A attractions *X*_12_	pcs	0.0646
			Total number of class 3A attractions *X*_13_	pcs	0.058
			National Wetland Park *X*_14_	pcs	0.0627
			Number of National Historic Cities *X*_15_	pcs	0.0692
			Number of cultural relics under state protection *X*_16_	pcs	0.0498
			Quantity of national intangible cultural heritage *X*_17_	pcs	0.0429
			Number of national / autonomous nature reserves *X*_18_	pcs	0.0851
			Number of National Forest Parks *X*_19_	pcs	0.088
		Public Facilities Resources	Total number of travel agencies *X*_20_	pcs	0.1212
			Number of Star Hotels *X*_21_	pcs	0.0683
			Highway density *X*_22_	km/km^2^	0.0463
			Paved road area *X*_23_	m^2^	0.0235
			Output value of logistics industry (parcel transportation, storage and post industry) *X*_24_	ten thousand yuan	0.0684
		Tourism Human Resources	Number of employees in the tertiary industry *X*_25_	pcs	0.0201
	TSC (weight 0.3420)	Social and Economic Support	Per capita disposable income of urban residents *X*_26_	yuan	0.0164
			Fixed asset investment of the whole society *X*_27_	ten thousand yuan	0.0899
			Utilization of foreign capital *X*_28_	ten thousand yuan	0.458
			Per capita Road area *X*_29_	m^2^	0.0378
		Ecological Environment Support	Days of good air quality *X*_30_	%	0.0507
			Harmless treatment capacity *X*_31_	tons per day	0.1908
			Urban water use penetration rate *X*_32_	%	0.0018
			Domestic waste treatment rate *X*_33_	%	0.0038
			Per capita park green area in urban area *X*_34_	m^2^	0.0531
			Green coverage of parks in the built-up area *X*_35_	%	0.0978

(Abbreviation: TC: Tourism Competitiveness; TDC: Tourism Development Competitiveness; TRC: Tourism Resource Competitiveness; TSC: Tourism-Support Competitiveness).

## 4. Analysis of the spatial difference of regional tourism competitiveness

The tourism development competitiveness, resource competitiveness, support competitiveness, and comprehensive competitiveness of 14 prefectures and cities in Xinjiang are assessed and divided into five levels, according to their scores: high- value areas, higher-value areas, moderate-value areas, lower-value areas, and low- value areas, using the entropy weighting method and the spatial analysis function of ArcGIS.

### 4.1. Tourism development competitiveness

**Fig 1:**
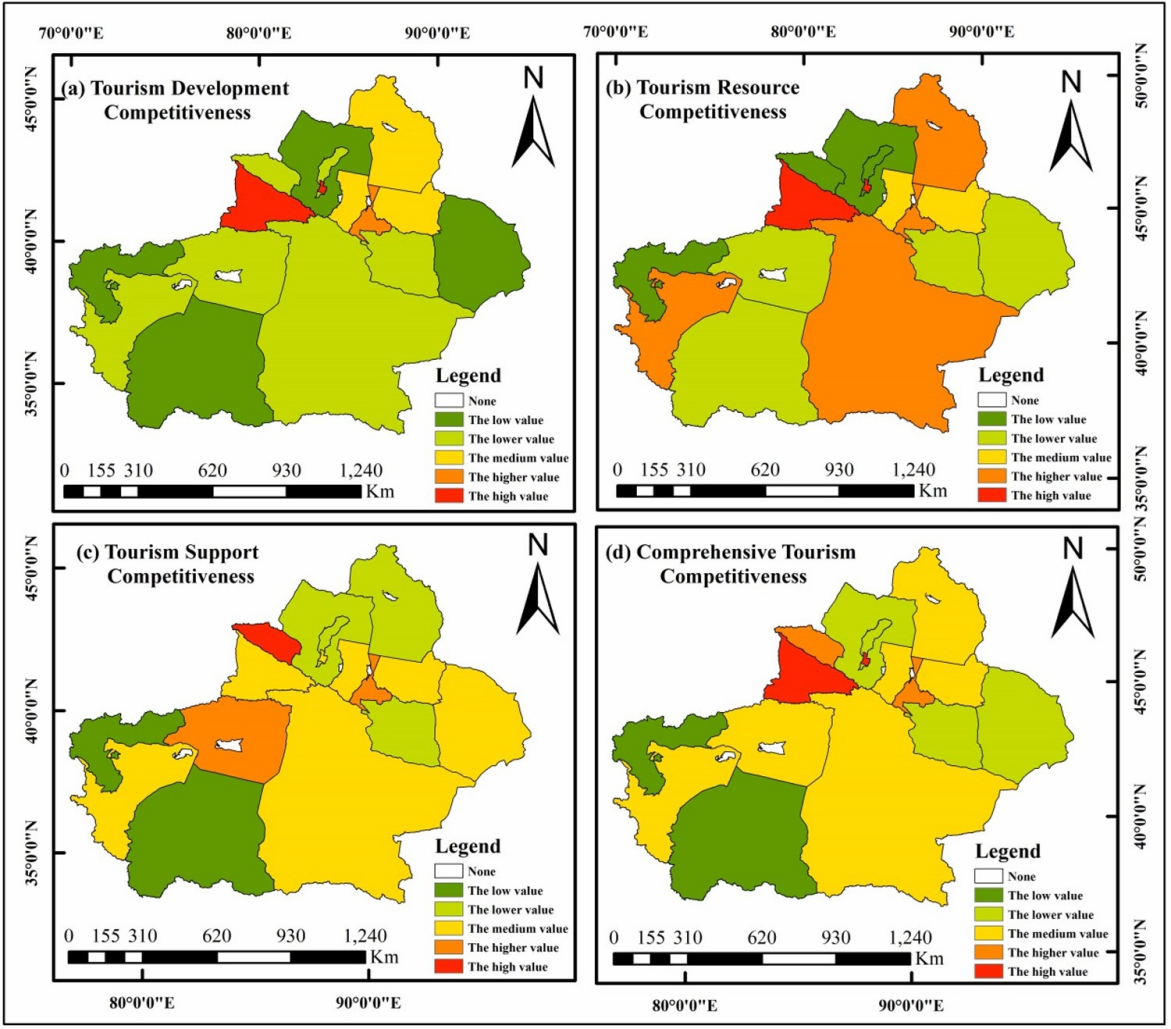
(a-d). Spatial difference analysis of tourism competitiveness in 14 regions of Xinjiang. Based on the results of tourism development competitiveness, resource competitiveness, support competitiveness, and comprehensive competitiveness, the ArcGIS spatial analysis function are used for classification, and the color indicates the strength of competitiveness. The blank area is the Xinjiang Production and Construction Corps, which is not within the scope of statistics. The base layer was downloaded from The Gateway to Astronaut Photography of Earth website (https://eol.jsc.nasa.gov/SearchPhotos/). Map credit: Haijun Liu.

Development competitiveness relates to regional economic development strength, tourism revenue, and tourism development ability, and it reflects the regional economic level and the development status of the tourism economy. The analysis of tourism development competitiveness (Fig 1A) indicates that there are obvious differences in tourism development competitiveness in Xinjiang. The score for tourism development competitiveness among counties and cities directly under the Yili Kazakh Autonomous Prefecture (Yili) is 0.6744, making it a high-value area; Urumqi City (0.3691) is a higher-value area; Changji Hui Autonomous Prefecture (Changji), with its score of 0.2041, Altay region (Altay) at 0.1518, and Bayingolin Mongolian Autonomous Prefecture (Ba Prefecture) with 0.1200, are moderate-value areas; The Kashi Region (Kashi), with its score of 0.1019, Karamay City at 0.0875, Aksu region (Aksu) with 0.0860, Turpan City (0.0853), Bortala Mongolian Autonomous Prefecture (Bo Prefecture) and its 0.0744, Tacheng region (Tacheng) at 0.0647, and Hami City (Hami) with 0.0700, are lower-value regions; and Hetian region (Hetian) has 0.0381, and Kizilsu Kirgiz Autonomous Prefecture (Ke Prefecture) has 0.0350, making them low-value regions. Thus, for competitiveness of tourism development, Yili and Urumqi are the centers, and Changji, Altay, and Ba Prefectures are secondary centers. Yili seized the opportunity to expand its tourism market, relying on the construction of China-Kazakhstan Horgos International Border Cooperation Zone. Yili put forward extensive effort to develop border tourism and port tourism, and also utilized the advantages of its natural ecological conditions to build a global tourism demonstration base and an international ecotourism valley. As the capital of the Xinjiang Uygur Autonomous Region, Urumqi has political, economic, cultural, and transportation advantages, but it does not have an obvious monopoly benefits, so it is inferior to Yili and is within a relatively high-value area. Changji and Altay are located in an economic belt at the north slope of Tianshan Mountain that has good traffic advantages and is also affected by the radiation effect of the core economic circle of Urumqi. The tourism development competitiveness of Changji and Altay is classified as median. Ba Prefecture is not only an important economic hub in southern Xinjiang but also a major tourist destination. The tourism market in Kashgar, Aksu, and Turpan is not commensurate with the resource advantages of these areas, and the tourism economic benefits are low there. Hetian and Ke Prefectures are located in a remote area of southern Xinjiang, their economic development is relatively lagging, and the distance index of tourist resources is relatively large. They are on the fringe of Xinjiang’s tourism market.

### 4.2. Tourism resource competitiveness

Resource competitiveness reflects the richness of regional tourism resources, and their attractiveness to tourists [62]. Fig 1B indicates that the competitiveness of regional tourism resources differs significantly across space. Yili (0.4870) is a high-value area, which is obviously superior to other regions with its rich tourism resources and a large number of A-level scenic spots. Urumqi (0.3272), Kashi (0.2970), Altay (0.2750), and Ba Prefecture (0.2730) are higher-value areas. Urumqi is a commodity trading, financial, transportation storage, and information service center in Western China and Central Asia. The Xinjiang International Grand Bazaar Scenic Area, Red Mountain Park, and Shuimogou Scenic Area are well-known in the territory. Kashgar was established as a special economic zone in 2010, It is a famous historical and cultural city well-known for its Uighur folk customs, culture and art, architectural style and traditional economy. The tourism resources of Altay are abundant. There are 48 A-level scenic spots, 3 National 5A scenic spots, 1 World Geopark, and 4 National Forest Parks. It is famous for Kanas Lake, the Kokoto Sea, Grassland Stone Man, and so on. Ba Prefecture is the largest administrative region at the prefecture level in China, with the famous scenic spots of Bayinbuluke Grassland, Bosten Lake, Ancient Loulan City, Lop Nur, Tarim Populus Euphratica Forest Park, and so on. In comparison, Urumqi does not have an advantage in tourism resources, but it is the political, economic, cultural, scientific, educational, financial, and transportation center of Xinjiang, It has perfect public facilities and rich human resources. Changji (0.2223), Turpan (0.1678), and Aksu (0.1516) are moderate-value areas. Hami (0.1229), Hetian (0.1213), Tacheng (0.1068), Bo Prefecture (0.1020), and Karamay City (0.0966) are lower-value areas, and Ke Prefecture (0.0542) is a low- value area. More than 90% of the territory is mountainous, with inconvenient transportation and a lack of high-quality tourism resources. Therefore, a spatial structure system of tourism resources competition is formed, with Yili as the core attraction area and Urumqi City, Kashi, Altay, and Ba Prefectures as the dominant attraction areas. These areas have abundant A-level scenic spots, key cultural relic protection units, intangible cultural heritages, natural reserves, and so on, which are high in value; further, they have other prominent resource advantages.

### 4.3. Tourism-support competitiveness

Tourism-support competitiveness includes social and economic support as well as ecological and environmental support, which reflects financial guarantees and environment bearing capacity to support regional tourism development [63, 64]. It can be seen from Fig 1C that the spatial pattern of regional tourism supports competitiveness: Bo Prefecture (0.5400) has a significantly higher value for tourism-support competitiveness than other regions; Urumqi (0.3040) and Aksu (0.2300) are higher-value regions; Hami (0.1260), Changji (0.1181), Ba Prefecture (0.1170), Yili (0.1160), Kashi (0.1080), and Karamay (0.1014) are moderate-value regions; Turpan (0.0914), Tacheng (0.0770), and Altay (0.0770) are lower-value areas; and Hetian (0.0610) and Ke Prefecture (0.0515) are low-value areas. In recent years, Bo Prefecture improved the financing mechanism, supporting facilities of reception services, and strengthened the comprehensive improvement of the tourism environment in the establishing and promotion of Sailimu Lake and Hot Spring National Tourism Resort. All of this enhanced its competitiveness in tourism support. Urumqi has improved the resilience of regional tourism by building an urban landscape belt and ecotourism demonstration area, improving the energy structure, and developing an environmental protection industry. Aksu is located at the northern edge of Taklimakan desert, with fragile ecological environment. In its development, Aksu has established the goal of creating a national forest city and the best investment environment in the west of China, and has vigorously implemented a number of projects to achieve this goal, such as protecting wetlands, greening urban roads, and enlarging public green space. At present, the coverage of the urban forest is 40.3%, and the per capita public green space area in Aksu is 9.2 square meters. It has won the titles of National Advanced City in Landscaping, National Health City, China Excellent Tourism City, and National Forest City. Except for the three regions mentioned above, the scores for tourism-support competitiveness in other regions are relatively low. Increasing investment, strengthening the management of scenic spots, enriching tourism infrastructure, and improving the basic tourism environment are all necessary for these regions.

### 4.4. Comprehensive tourism competitiveness

The comprehensive competitiveness of tourism is assessed through a weighted summation of the tourism subsystem competitiveness of 14 prefectures and cities in Xinjiang (Fig 1D). The spatial distribution map of comprehensive tourism competitiveness shows that in Xinjiang, the high-value area for regional tourism competition is located in Yili (0.4405), and the higher-value areas are Urumqi (0.3373), and Bo Prefecture (0.2400); the moderate-value areas are Changji (0.1790), Altay (0.1580), Ba Prefecture (0.1538), Aksu (0.1500), and Kashi (0.1484); the lower- value areas are located in Turpan (0.1058), Hami (0.0993), Karamay (0.0943), and Tacheng (0.0785); and the low-value areas are Hetian (0.0649) and Ke Prefecture (0.0450). In a word, the spatial distribution of Xinjiang’s comprehensive tourism competitiveness takes Yili as the center and Urumqi and Bo Prefecture as the subcenters; Changji, Altay, Ba Prefecture, Aksu, and Kashi constitute a multi-polar advantage competition area structure. The spatial characteristics of polarization, diffusion and geospatial nearest neighbor effect are obvious, showing banded and circular distribution and a decreasing trend from the center to the surrounding areas in general. Regarding the development mode, it is more inclined to the structural mode of the polar core and point axis.

## 5. Influencing factors of spatial differences in tourism competitiveness

Using factor analysis, the feasibility of a standardized data approach to index factors is tested. The KMO statistic is 0.834, greater than 0.7, the threshold. This shows that the degree of overlap in information among the variables is high, which can be reduced by factor analysis. The value for Bartlett’s sphericity test of 381.382, the degree of freedom (*df*) of 15, and the significance level (*sig*.) of 0.000 in the case of rejection of the hypotheses of the independent variables, shows that a strong correlation among variables, and the samples are thus suitable for factor analysis. The maximum variance rotation method was used to polarize the square value of the load factor to 0 and 1, which makes the results of the analysis more obvious. Following the principle that the eigenvalue is greater than 1, the cumulative variance contribution rate of the eight extracted eigenvalues is 92.714% (Table 2), indicating that the common factors extracted retain most of the information in the original data and greatly reduce its complexity. By analyzing the component rotation load matrix of the eight common factors, the influencing factors for regional tourism competitiveness in Xinjiang can be understood to be socio-economic condition, tourism resources, infrastructure construction, regional cultural influence, ecological environment bearing capacity, tertiary industry development, tourism service level, and living security system.

**Table 2:** Factor eigenvalue and cumulative variance contribution rate.

Components	Initial eigenvalue			Rotate square sum load		
	Total	Variance%	Accumulate %	Total	Variance %	Accumulate %
1	13.139	37.539	37.539	10.843	30.980	30.980
2	5.361	15.316	52.854	6.133	17.524	48.504
3	3.563	10.179	63.033	3.474	9.926	58.430
4	3.189	9.110	72.144	3.156	9.017	67.446
5	2.600	7.428	79.571	2.660	7.601	75.048
6	1.893	5.408	84.979	2.358	6.737	81.785
7	1.378	3.936	88.915	2.094	5.984	87.769
8	1.330	3.799	92.714	1.731	4.945	92.714

### (1) Social and economic conditions

The initial eigenvalue and cumulative contribution rate for the first common components are 13.139 and 30.980% (Table 2). *X*_2_, *X*_3_, *X*_4_, *X*_5_, *X*_9_, *X*_20,_ and *X*_24_ are significant in the rotation load matrix, and their values of their variables are 0.944, 0.869, 0.862, 0.941, 0.856, 0.953, and 0.930 respectively (Table 3). Among these, domestic tourism revenue (*X*_2_), GDP (*X*_5_), and logistics output value (*X*_24_) reflect regional economic strength. A good economic foundation can effectively promote tourism development. The number of guest rooms (*X*_3_), the number of guest beds (*X*_4_), the number of domestic tourists (*X*_9_), and the total number of travel agencies (*X*_20_) reflect the degree of achievement of tourism public facilities, and this is an important ensuring orderly development of tourism activities. This factor is defined as socio-economic conditions, indicating the comprehensive characteristics of tourism.

**Table 3:** Component rotating load matrix.

Variables	Components							
	1	2	3	4	5	6	7	8
*X*_1_	0.565	0.273	0.010	0.297	0.503	0.023	-0.353	-0.278
*X*_2_	0.944	0.126	-0.027	-0.016	0.133	0.218	-0.092	-0.062
*X*_3_	0.869	0.431	-0.006	0.177	0.095	-0.052	-0.087	0.052
*X*_4_	0.862	0.430	-0.001	0.189	0.119	-0.049	-0.108	0.049
*X*_5_	0.941	0.054	-0.061	-0.016	-0.076	-0.219	0.195	0.011
*X*_6_	0.200	-0.487	0.539	-0.350	0.211	0.175	0.326	0.090
*X*_7_	0.008	0.047	-0.067	-0.053	-0.099	0.970	0.108	0.009
*X*_8_	0.662	0.260	-0.029	0.221	0.474	0.003	-0.340	-0.252
*X*_9_	0.856	0.188	0.058	0.014	0.126	0.393	0.068	0.025
*X*_10_	0.369	0.837	-0.010	0.091	-0.062	0.250	0.134	-0.012
*X*_11_	0.358	0.747	0.349	0.182	0.097	0.006	0.005	-0.046
*X*_12_	0.777	0.230	0.045	0.393	0.224	0.091	-0.211	0.107
*X*_13_	0.298	0.787	0.251	-0.070	-0.064	0.248	0.147	-0.170
*X*_14_	0.280	0.889	-0.095	0.105	0.225	0.074	-0.054	-0.186
*X*_15_	-0.080	0.089	0.427	0.760	-0.261	0.051	-0.058	-0.113
*X*_16_	0.208	0.091	0.057	0.945	0.056	-0.079	-0.089	0.005
*X*_17_	0.413	0.760	-0.151	0.253	-0.201	-0.247	0.176	-0.088
*X*_18_	0.033	0.390	-0.046	0.241	0.763	-0.292	0.047	0.127
*X*_19_	0.456	0.556	0.173	-0.038	0.368	0.463	-0.224	-0.026
*X*_20_	0.953	0.153	-0.073	-0.175	-0.088	-0.050	0.027	0.010
*X*_21_	0.757	0.410	0.063	0.328	0.284	0.027	-0.224	-0.062
*X*_22_	0.694	-0.216	0.005	0.169	0.064	-0.085	-0.155	-0.515
*X*_23_	-0.201	0.082	0.909	0.199	-0.031	-0.109	-0.155	-0.036
*X*_24_	0.930	0.076	-0.049	-0.018	-0.101	0.185	0.255	0.018
*X*_25_	0.018	0.162	-0.214	-0.110	-0.001	0.135	0.924	-0.002
*X*_26_	0.452	-0.595	0.435	-0.305	0.061	0.021	0.277	0.208
*X*_27_	0.723	0.254	-0.047	0.119	-0.131	0.510	0.312	0.050
*X*_28_	-0.157	-0.261	-0.276	-0.221	0.422	-0.016	-0.066	-0.125
*X*_29_	-0.201	0.082	0.909	0.199	-0.031	-0.109	-0.155	-0.036
*X*_30_	0.064	-0.169	0.255	-0.400	0.789	0.111	0.082	0.081
*X*_31_	0.795	-0.138	-0.076	-0.313	-0.366	-0.059	0.244	0.109
*X*_32_	-0.059	-0.377	-0.033	-0.451	-0.017	0.156	-0.379	0.679
*X*_33_	0.315	0.169	0.672	-0.098	0.202	0.428	-0.007	-0.172
*X*_34_	-0.184	0.692	0.369	-0.220	0.114	-0.146	0.052	0.401
*X*_35_	0.372	-0.290	-0.312	0.293	0.081	-0.175	0.224	0.674

### (2) Occurrence of tourism resources

The characteristic root of component 2 is 5.361, and the cumulative variance contribution rate is 52.854% (Table 2). *X*_10_, *X*_11_, *X*_13_, *X*_14,_ and *X*_17_ are significant in the second component rotation load matrix, with the values of 0.837, 0.747, 0.787, 0.889, and 0.760, respectively (Table 3). This factor reflects the quantity and grade of regional tourism resources. Xinjiang is favored by consumers for its colorful natural landscape and simple and straightforward customs. At present, it features 12 5A level scenic spots, 109 4A level scenic spots, 287 3A level scenic spots, 48 national wetland parks, 113 national cultural relics protection units and 371 national intangible cultural heritages. This region has the most abundant tourism resources in China to meet the needs of different types of tourism consumption [65].

### (3) Infrastructure construction

In the third component, the variables with high correlation are *X*_23_ and *X*_29_, the characteristic root of the common factor is 3.563, the contribution rate of cumulative variance is 63.033% (Table 2), and the value of the rotary load factor is 0.909 (Table 3). The area of paved road and the area per capita of paved road reflect the supply of reliable tourism infrastructure, which are a requirement for tourists to be able to do tourism activities at the destination. During the 12th Five Year Plan period, the region increased its construction of tourism infrastructure and public services system, 1 353 new tourism projects were added, and the fixed asset investment in railway, highway, and civil aviation was about 270 billion yuan. A comprehensive transportation network pattern based on the trunk railway and expressway, national and provincial trunk and branch railways, and rural highways was initially formed, and a complete transportation system ensured the rapid development of tourism in Xinjiang [66–68].

### (4) Regional cultural influence

The characteristic root for factor 4 is 3.189, and the cumulative variance contribution rate is 72.144% (Table 2). *X*_15_ and *X*_16_ are significant for the rotation load matrix, and their variable values are 0.760 and 0.945, respectively (Table 3). Xinjiang is an important channel for the ancient Silk Road and a link in the convergence between Chinese and Western civilizations. It features rich cultural relics and diverse cultures. Among these 22 famous historical and cultural cities, 113 national cultural relics protection units, and 371 national intangible cultural heritages. During the 13th Five Year Plan period, Xinjiang’s tourism development has adopted a focus on the One Belt, One Road strategy, seizing the opportunity to obtain counterpart assistance to Xinjiang. Xinjiang was able to make great efforts to build a tourism distribution center for the Silk Road Economic Belt and a tourist destination for Silk Road culture and ethnic customs in southern Xinjiang.

### (5) Bearing capacity of ecological environment

The characteristic root for factor 5 is 2.600, and the cumulative variance contribution rate is 79.571% (Table 2). *X*_18_ and *X*_30_ have high correlation, with load factor values of 0.763 and 0.789 (Table 3). The number of national or autonomous nature reserves and the number of days of good air quality reflect the regional ecological environment. The development of tourism is easily affected by the climate and the local natural environment. The bearing capacity of the ecological environment determines whether the regional tourism industry can be sustainably developed. According to the current motto in Chinese government in this area, namely, “green water and green mountains are golden mountains and silver mountains”, eco-friendly tourism products should be developed, low-carbon tourism and green consumption should be advocated, and an ecological civilization tourism concept should be built.

### (6) Development of tertiary industry

*X*_7_ is more significant in its sixth component, the value of its load factor is 0.970 (Table 3), and its initial characteristic value and cumulative contribution rate of the common factor are 1.893 and 84.979%, respectively (Table 2). The tertiary industry is an important indicator for measuring the modernization level of the national economy of a region. It can effectively stimulate market vitality; guide the free flow of capital, technology, and talent in different industries and departments, and provide favorable external environment for the development of tourism. For example, Urumqi does not have an advantage in the influence and monopoly of tourism resources, but the tertiary industry in Urumqi accounts for 70.22% of its GDP, which endows it with the strong advantages of economic attraction, perfect infrastructure and high-quality tourism service ability. All of this makes Urumqi an important tourism market for Xinjiang.

### (7) Tourism service level

The characteristic root of factor 7 is 1.378, and its cumulative variance contribution rate is 88.915% (Table 2). *X*_25_ is more significant for the rotating load matrix, and its load factor value is 0.924 (Table 3). Tourism service level refers to the service function, service quality and service efficiency embodied in the tourism service complex. Tourism service level involves tourism infrastructure, tourism product quality, tourism environment quality, destination image, employee quality, tourism service management, which runs through the whole link of tourism activities, and is the guarantee to support the sustainable development of tourism industry. The Tourism 13th Five Year Plan clearly called for further promotion of the construction of the Global Tourism and Internet Plus Tourism-support platforms, making full use of the internet, the Internet of Things, Big Data, and cloud platforms to promote integration and innovation in the tourism industry, and continuing to improve the software and hardware facilities of tourism services, striving to develop Xinjiang’s tourism into a strategic pillar industry to stabilize the territory and enriching the people.

### (8) Living security system

*X*_32_ and *X*_35_ are significant for the eighth component, and their rotation load factor values are 0.679 and 0.674, respectively (Table 3). The factor characteristic root is 1.330, and the cumulative variance contribution rate is 92.714% (Table 2). The tourism industry has a high degree of relevance, and the most basic prerequisite for the development of tourism is meeting the living needs of tourists in all aspects of their tourism activities [69]. The improvement of the regional living security system will directly affect tourists’ perception of the destination.

## 6. Discussion

The measurement of tourism competitiveness requires systematic and comprehensive work. Many factors affect tourism development. Different research methods cause the weighting of index factors and results of evaluation to often deviate from the actual situation [70]. In recent years, the study of tourism competitiveness has mostly adopted a comprehensive factor model. Competitiveness is a multielement concept, involving location conditions, spatial accessibility, resource availability, economic development conditions, market potential, infrastructure, human resources, and government management [71–73]. The composition of the evaluation index system and decomposition of the target level, as well as the quantification and distribution of index weights, are all difficult problems. This study comprehensively considers the construction of index system in terms of multiple dimensions, such as the economy, society, and ecology; it adopts an objective weighting method for data calculation to avoid the interference of subjective weighting on the research results.

In this study, as the capital of Xinjiang Uyghur Autonomous Region, Urumqi has political, economic, cultural, transportation, and other advantages, but it is second to Yili in terms of tourism competitiveness, indicating that Xinjiang’s tourism industry is still in a resource-oriented stage. This is inconsistent with China’s provincial-scale market-oriented tourism competition pattern [74, 75]. This finding is consistent with Zhenting Li’s research on the validity of tourism competitiveness in China’s provincial tourism areas, tourism competitiveness is a dynamic variable, and its value changes with research scale and over time [76].

Tourism resources, economic conditions, and development potential vary in different regions. To determine an evaluation system for tourism competitiveness in the research area, we should focus on horizontal comparison within the region and also take into account vertical comparisons across periods to grasp the development potential and future trends of the regional tourism industry as a whole. In addition, the evaluation system for regional tourism competitiveness forms part of a system of tourism development competitiveness, resource competitiveness, and support competitiveness. In addition to single-factor differences, it is necessary to consider changes in these factors over time. Moreover, this study of the evaluation of tourism competitiveness and spatial differentiation mechanisms in Xinjiang does not involve the tourists’ own behavior, tourism satisfaction, tourism market mechanisms, policies, or other factors, which require further exploration and analysis in the future.

## 7. Conclusion

This study constructs an evaluation index system for regional tourism competitiveness in relation to the three aspects of development competitiveness, resource competitiveness, and support competitiveness. It uses the entropy weight method and spatial analysis function of ArcGIS to evaluate the tourism subsystem comprehensive competitiveness of 14 regional subdivisions of Xinjiang and explores the driving mechanisms of their spatial differentiation of tourism competitiveness through the factor analysis method.

Yili is found to be a high-value area in terms of competitiveness of tourism development; Urumqi City is a higher-value area; Changji, Altay, and Ba Prefecture are moderate-value areas; Kashi, Karamay, and Aksu are lower-value areas; and Hetian and Ke Prefecture are low-value areas. The overall development pattern takes Yili and Urumqi to be the center and Changji, Altay, and Ba Prefecture to be the secondary centers. Regarding competitiveness of tourism resources, Yili is obviously superior to the other regions considered and is a high-value area; Urumqi, Kashi, Altay, and Ba Prefecture are higher-value areas; Changji, Turpan, and Aksu are moderate-value areas; Hami, Hetian, Tacheng, Bo Prefecture, and Karamay are lower-value areas; and Ke Prefecture is a low-value area. Thus, a spatial structure system is formed of competition for tourism resources, with Yili as the core attraction area, and Urumqi, Kashi, Altay, and Ba Prefecture as the dominant attraction areas. The spatial pattern of tourism-support competitiveness is as follows: Bo Prefecture is a high-value area; Urumqi and Aksu are higher-value areas; Hami, Changji, and Ba Prefecture are moderate-value areas; Turpan, Tacheng, and Altay are lower-value areas, and Hetian and Ke Prefecture are low-value areas. The high-value area of comprehensive tourism competitiveness is limited to Yili; the higher-value areas are Urumqi and Bo Prefecture; and the moderate-value areas are Changji, Altay, Ba Prefecture, Aksu, and Kashi. Lower-value areas are distributed across Turpan, Hami, Karamay, and Tacheng, and Hetian and Ke Prefecture are low-value areas. Thus, Yili is the center, Urumqi and Bozhou are subcenters, and Changji, Altay, Ba Prefecture, Aksu, and Kashi are multi-polar competitive areas.

Using the KMO and the Bartlett sphericity tests, the cumulative variance contribution rate of eight factor characteristic roots extracted by the maximum variance rotation method is 92.714%, and the driving mechanism of the spatial differentiation of tourism competitiveness in Xinjiang can be assessed as follows: socio-economic conditions, tourism resources, infrastructure construction, regional cultural influence, ecological environment carrying capacity, tertiary industry development, tourism service level, and living security system. For the first component (social and economic conditions), domestic tourism revenue, GDP, logistics output value, and other variables are the most significant. The total number of A-level scenic spots, the National Wetland Park, and national intangible cultural heritages are more significant in the second component (occurrence of tourism resources). The area of paved roads is more significant in the third component (infrastructure construction). In the fourth component (regional cultural influence), national historical cultural cities and national cultural relic protection units are important and reflect the influence of regional culture. The number of national and regional nature reserves and the number of days with good air quality are highly correlation in the fifth component (bearing capacity of ecological environment). The proportion of tertiary industry in terms of GDP and the number of employees working in tertiary industry are significant for the sixth (development of tertiary industry) and seventh components (tourism service level), respectively. In the eighth component (living security system), a high correlation is seen between the urban water utilization rate and the green coverage rate of parks in built-up areas.

## Supporting information

S1 Data (XLSX)

S2 Data (XLSX)

S3 Data (XLSX)
